# Prevalence of opioid misuse in patients with cancer: a systematic review and meta-analysis

**DOI:** 10.1038/s41416-024-02802-8

**Published:** 2024-07-26

**Authors:** Tazha Ako, Mark Puch Ørnskov, Camilla Lykke, Per Sjøgren, Geana Paula Kurita

**Affiliations:** 1 Faculty of Health and Medical Sciences, University of Copenhagen, Copenhagen, Denmark; 2 Department of Clinical Medicine, Faculty of Health and Medical Sciences, University of Copenhagen, Copenhagen, Denmark; 3 Multidisciplinary Pain Centre, Department of Anesthesiology, Pain and Respiratory Support, Rigshospitalet Copenhagen University Hospital, Copenhagen, Denmark; 4 Section of Palliative Medicine, Department of Oncology, Rigshospitalet Copenhagen University Hospital, Copenhagen, Denmark; 5 Department of Oncology and Palliative Care, North Zealand Hospital, Hillerød, Denmark

**Keywords:** Cancer, Drug regulation, Pain

## Abstract

**Background/Objectives:**

Long-term consequences of opioid consumption, such as misuse, have been a major concern in patients with chronic non-cancer pain. Potentially opioid misuse may also be a consequence in patients with cancer in opioid treatment which encouraged us to undertake this systematic review assessing the frequency of opioid misuse in this population.

**Materials/Methods:**

The search strategy comprised words related to cancer, opioid misuse, and frequency. PubMed, Embase, PsycInfo, and Cinahl were searched from inception to July 2023. Prospective studies were selected and analysed regarding frequency, study characteristics, and quality. A meta-analysis was possible to carry out for a sub-group (opioid misuse risk).

**Results:**

From 585 abstracts screened, six articles were included. Only prevalence data were found. The prevalence of opioid misuse ranged from 5.7% to 84%, while the prevalence of opioid misuse risk varied from 2.4% to 35.4%. The pooled prevalence of opioid misuse risk was 12.3% (95% CI: 0.8–36.3; *I*^2^ = 98.4%, 95% CI: 97.2–99.1). The studies differed regarding, e.g., methods, misuse definitions, and assessment instruments.

**Conclusions:**

Few studies were identified and large differences in prevalence for opioid misuse and opioid misuse risk were observed. Methodological disparities and the studies quality underscore the importance of improved studies in the future.

## Introduction

Cancer is a complex and life-threatening disease, which according to estimates will affect 21.9 million people around the world in 2025 [1]. In patients with cancer and especially advanced disease, pain is one of the most frequent and distressing symptoms [2, 3]. It might be the result of the cancer disease itself and/or antineoplastic treatment. The clinical evidence supports the use of opioids as the gold standard in moderate to severe cancer-related pain [4]. In addition, opioids are a versatile pharmacological group with several advantages as superior efficacy, multiple routes of administration, ease of titration, no ceiling dose, among others. However, in the last two decades the use of opioid treatment in chronic non-cancer pain has produced an indiscriminate escalation of prescribed opioids and misuse problems, which recently have raised concerns about potential misuse in patients with cancer, mainly due to higher survival rates.

In the literature, there are many different terms with similar meanings and referring to types of opioid misuse. Examples are opioid dependence, opioid use disorder, opioid abuse, opioid addiction, opioid aberrant behaviour, non-medical opioid use, and chemical coping, among others. There are general diagnostic criteria for opioid dependence by the International Classification of Diseases—ICD 11 [5] and for opioid use disorder by Diagnostic and Statistical Manual of Mental Disorders fifth edition—DSM-5 [6], which describe behaviour, psychological aspects, and physiologic symptoms. For patients with chronic pain, there are Portenoy’s criteria for characterising opioid addiction [7] and Chabal et al.’s criteria for identifying prescription opiate abuser [8], which are based on the general criteria mentioned above and describe behaviours and factors indicating a diagnosis. According to the National Institute on Drug Abuse (USA) opioid misuse can be defined as the use of prescription opioids in a manner other than as directed by a doctor (e.g., for other purpose than pain relief, in greater amounts, more frequently, longer, using someone else’s prescription) [9]. This definition comprises the concepts of opioid dependence, opioid abuse, opioid use disorder, opioid addiction, and aberrant opioid behaviour. Thus, the term opioid misuse and its operational definition above is preferred in this study.

Advances in diagnostics and antineoplastic treatments have improved prognosis and increased survival in patients with cancer. Population based analyses of high-income countries have indicated that 3 to 5-year relative survival has improved for most cancers [10]. A decrease in the American cancer death rate of 33% has been observed from 1991 to 2020, for instance [11, 12]. Survivors may suffer the consequences of the disease and treatment. Approximately 40–45% of the patients with cancer and 35% of the cancer survivors have chronic pain [2, 13]. Therefore, it is expected that many patients will be treated with opioids and may continue using it for long periods even after concluding cancer treatment.

Despite the legitimate medical use of opioids in cancer pain, there is concern about several potential consequences of their prolonged use as development of endocrine and immune systems suppression, neurotoxicity, tolerance, physical and psychological dependence including opioid misuse [14–17]. In particular, the lessons learned from the opioid crisis in the United States have raised an alert regarding increasing consumption of prescribed and illegal opioid drugs associated with overdoses and deaths. The American numbers of drug overdose deaths involving opioids have shown alarming increasing rates since 1999. In 2022, 3/4 of the drug overdose deaths (107,000) included an opioid [18].

The prevalence of opioid misuse in patients with chronic non-cancer pain has been reported between 3.2 and 80.5% [19]. Obviously, there is also a question whether it would also be observed in patients with cancer in a changing environment. Previous systematic reviews about the theme in patients with cancer have also reported a varied prevalence between 2.0 and 90.0%, but the studies analysed had high heterogeneity regarding designs, mixed populations, type of substance use (not always specific for opioids), operational definitions for the opioid misuse, and included studies published until 2020 [20–22]. Thus, the need for an updated assessment of the literature regarding frequency of opioid misuse in the cancer population was deemed necessary. In summary, this systematic review aimed at assessing the frequency of opioid misuse and opioid misuse risk in patients with cancer in all stages. Hopefully, the information generated by this study can help to reduce the gap in knowledge on this subject and point directions for future studies. Through this endeavour, we hope to contribute to improved patient outcomes and enhance the overall quality of cancer care.

## Materials and methods

This systematic review was conducted following the Preferred Reporting Items for Systematic Reviews and Meta-Analyses—PRISMA guidelines [23] to answer the research question: ‘In adult patients with cancer, what is the frequency of opioid misuse?’ A comprehensive literature search was performed to identify studies that investigated the frequency of opioid misuse in patients with cancer, adhering to the predetermined research question, search strategy and inclusion and exclusion criteria.

### Search strategy

To identify relevant studies on the prevalence of opioid misuse in patients with cancer, the databases PubMed, Embase, Cinahl, and PsycInfo were systematically searched from inception to 14 July 2023. The search terms were structured based on PICO framework, which focus on the Population, Intervention, Comparison and Outcomes [24, 25]. For this review, the search strategy included the elements cancer patients (population) and frequency of opioid misuse (outcomes), which were organised in three groups of words/terms. Search limits (filters) of the databases were used to optimise the search considering the identification of key words/terms in titles and abstracts. The final search strategy was organised as follow:

(((cancer[Title/Abstract] OR neoplasms[Title/Abstract]) AND (non-medical opioid use[Title/Abstract] OR opioid use disorder[Title/Abstract] OR opioid abuse[Title/Abstract] OR opioid addiction[Title/Abstract] OR opioid misuse[Title/Abstract] OR substance use disorder[Title/Abstract] OR opioid dependence[Title/Abstract] OR opioid aberrant behaviour[Title/Abstract] OR opioid aberrant behaviour[Title/Abstract] OR chemical coping[Title/Abstract] OR problematic opioid use[Title/Abstract])) AND (prevalence[Title/Abstract] OR frequency[Title/Abstract] OR occurrence[Title/Abstract] OR incidence[Title/Abstract])).

### Selection criteria

The retrieved abstracts were read by two independent reviewers (TA and GPK). They selected potential articles to analysis, according to the pre-established criteria:

Inclusion criteria: studies involving adults (at least 18 years old) with cancer; prospective studies; studies focusing on opioid treatment for cancer-related pain management; studies reporting the frequency or risk of opioid misuse among patients with cancer; studies that employed a standardised assessment of opioid misuse; and articles written in English.

Exclusion criteria: studies with mixed populations without separate results for the individuals with cancer; abstracts of studies presented in scientific events; and study protocols.

### Screening and extraction

Abstracts from each literature search in the different databases were imported into Covidence, which is a software for managing and streamlining systematic reviews (https://www.covidence.org/). Duplicated abstracts were excluded via the programme. Two individuals (TA and GPK) have individually screened the abstracts and read the full text of the selected ones. Disagreements between readers were resolved through discussion between them. No hand-search was performed. The same two individuals extracted the data independently and compared them together.

### Analysis

The main outcome of this study was the frequency of opioid misuse and opioid misuse risk, according to definition described previously: any use of opioids not as prescribed by the physician. Observing that studies might have assessed diverse aspects of opioid misuse (e.g., risk for opioid misuse), they could be grouped according to similar outcomes described. The frequency information was collected in absolute and percentual numbers.

The quality assessment of the studies was performed according to the Joanna Briggs Institute critical appraisal checklist for studies reporting prevalence data [26]. The checklist includes nine questions regarding study features that could increase or reduce the consistency of the evidence. The possible answers for each item are yes, no, unclear, and not applicable. Considering the possibility that some studies could not present enough information according to the checklist, some items were adapted. A summarised description of the aspects considered essential for analysing the quality is given in the following. (1) Was the sample frame appropriate to address the target population? Appropriateness of the sample frame to address the target population by looking at the broader characteristics, demographics, and medical history to avoid making inferences that might not represent the target population. (2) Were study participants sampled in an appropriate way? Appropriate sampling methods with consideration for the representativeness of the base population. Convenience samples do not provide a representative sample. (3) Was the sample size adequate? Information about sample size should be provided in the article to analyse the precision of the final estimate. (4) Were the study subjects and the setting described in detail? Subjects and settings should be described enough to ensure comparability with the population of interest. (5) Was the data analysis conducted with sufficient coverage of the identified sample? Adequate representation of all subgroups within the sample, adequate response rate. (6) Were valid methods used for the identification of the condition? Outcomes assessed based on existing definitions/diagnostic criteria. (7) Was the condition measured in a standard, reliable way for all participants? Use of standardised and reliable measurement methods for assessment of the condition in all participants. (8) Was there appropriate statistical analysis? Enough information about proper analytical technique used and how variables of interest were measured. (9) Was the response rate adequate, and if not, was the low response rate managed appropriately? Information about response rate, reasons for non-response, characteristics comparison between responders and non-responders.

### Statistical analysis

Studies that have assessed the same outcomes related to opioid misuse with the same instrument were selected for meta-analysis. A random-effects model was applied due to the heterogeneity among studies and their individual effect sizes. Pooled prevalence of moderate and high risk of opioid misuse with 95% confidence intervals (CI) and heterogeneity (Cochran’s *Q* test and *I*^2^ statistics) were analysed using MedCalc v.22.009 (MedCalc Software Ltd). *I*^2^ values range from 0 to 100% and larger values mean increasing heterogeneity. Attempt of cut-offs are 25% = low, 50% = moderate, and 75% = high [27].

## Results

### Search result

**Fig. 1:**
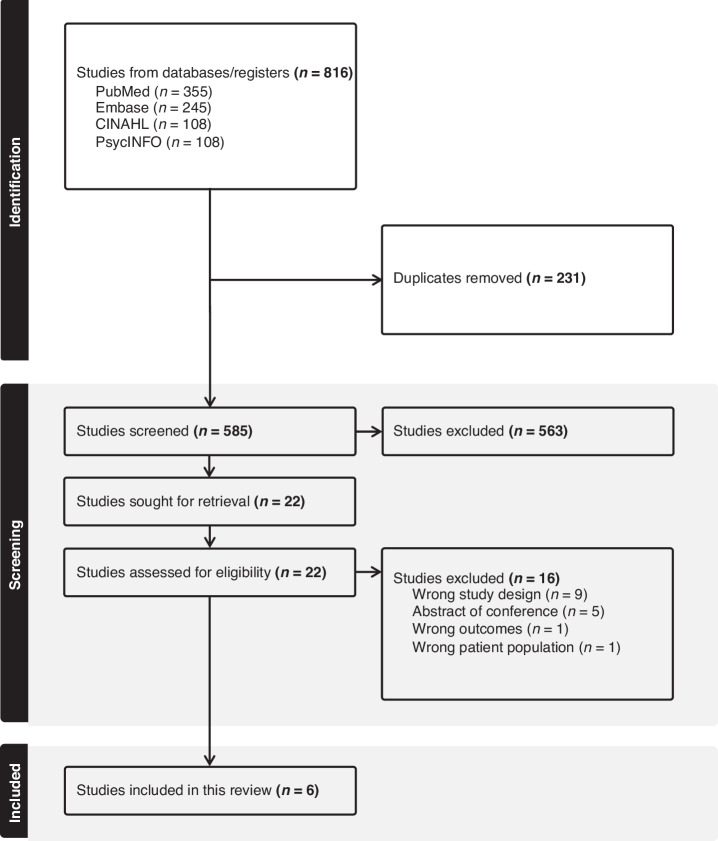
Flow diagram of screening process and study selection.

The database search comprised 816 studies. The number of duplicates removed was 231. Therefore, 585 abstracts were screened; out of them, 563 were excluded. Twenty-two articles were selected for full reading and inclusion and exclusion criteria were reapplied. Finally, six studies were included in the analysis. Reasons for studies exclusion after full reading can be observed in Fig. 1.

### Characteristics of the selected studies

The six studies analysed reported prevalence and were divided in equal numbers in two groups according to the outcomes evaluated: prevalence of opioid misuse [28–30] or prevalence of opioid misuse risk [31–33]. Two studies were conducted in the United States [29, 31], the others were from Iran [28], Italy [32], Japan [30], and South Korea [33]. All of them had a cross-sectional design but differed regarding other characteristics (Table 1). Five studies were conducted in patients with cancer disease [28, 29, 31–33], one study analysed the general population with chronic pain, mixing cancer and non-cancer conditions [30].

**Table 1:** Studies’ characteristics and opioid misuse prevalence.

Author, ref. country	Study design	Sample	*N*	Age in years mean/range	Duration opioid treatment	Dose opioid^a^	Opioid misuse type	Prevalence *n* (%)	Assessment instrument
Misuse									
1. Tabei et al. [28] Iran	Cross-sectional	Gastric cancer patients	177	58.1/24–84	–	–	Opioid dependence	10 (5.7)	Interview based on DSM-IV criteria
2. Kwon et al. [29] United States	Cross-sectional	Advanced cancer patients	432	–/49–65	–	Median 100(38–225)	Chemical coping	76 (17.6)	Medical assessment
3. Takasusuki et al. [30] Japan	Cross-Sectional (internet survey)	General population with chronic pain (sub-group chronic cancer pain)	61	53.0/–	87% ≥3 months	–	Opioid misuse/abuse	51 (84.0)	Survey questions
Moderate/severe misuse risk									
4. Dannenberg et al. [31] United States	Cross-sectional	Solid or liquid tumours patients	226	63.7/28–80+	–	–	Opioid misuse risk	63 (28.0)	Opioid Risk Tool
5. Mercadante et al. [32] Italy	Cross-sectional	Advanced cancer patients	113	68/–	≥7 days	–	Opioid misuse risk Aberrant opioid behaviour prediction	14 (12.3) 40 (35.4)	Opioid Risk Tool Screener and Opioid Assessment for Patients with Pain
6. Oh et al. [33] South Korea	Cross-sectional	Cancer-related pain patients	946	63/–	≥7 days	Mean 93.1 ± 246.8	Opioid misuse risk	23 (2.4)	Opioid Risk Tool

– no information, *DSM-IV* Diagnostic and Statistical Manual of Mental Disorders—4th edition.

^a^Morphine equivalent daily dose in mg.

The studies differed regarding sample size, definitions used to characterise opioid misuse, recruitment methods, assessment format and instruments of assessment (Table 1). In particular, the assessment of opioid misuse was made through pre-defined criteria as DSM-IV criteria [28] and operational definitions [29, 30]. All studies in the group of prevalence of opioid misuse risk applied instruments developed for assessment of chronic non-cancer pain patients. The instruments were the Opioid Risk Tool [31–33] and the Screened and Opioid Assessment for Patients with Pain [32] (Table 1). Only the second instrument has been validated for use in cancer patients.

Missing Information regarding accurate duration of opioid administration (3/6), daily opioid dose (4/6), and distribution of type of opioid used (6/6) was observed (Table 1).

### Prevalence of opioid misuse and meta-analysis of moderate and high risk of opioid misuse

The prevalence of opioid misuse reported varied between 5.7 and 84% (Table 1), while the prevalence of opioid misuse risk varied between 2.4 and 35.4% (Table 1). One study assessed the prevalence through two different instruments, which resulted in contrasting percentages as 12.3 and 35.4% [32]. In addition, in the same study clinical staff was instructed to report any aberrant opioid behaviour as part of their clinical routine within a month from the assessment; 74% of the patients returned for follow-up and no opioid misuse was registered [32].

**Fig. 2:**
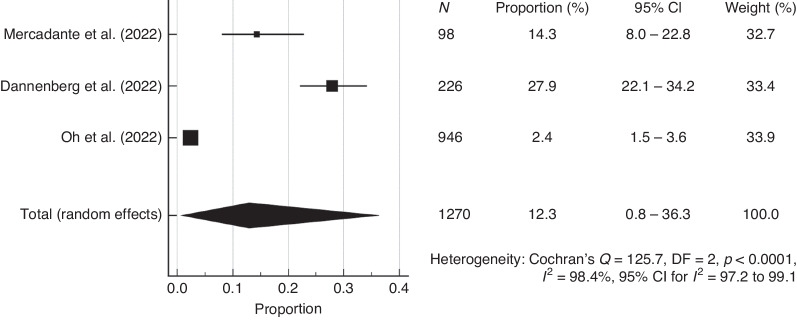
Pooled prevalence of moderate and high risk of opioid misuse.

For the studies that applied the same questionnaire, the pooled prevalence of moderate and high risk of opioid misuse can be observed in Fig. 2. The pooled prevalence was 12.3% (95% CI: 0.8–36.3) and heterogeneity *I*^2^ = 98.4% (95% CI: 97.2–99.1).

### Quality assessment of included studies

One study contemplated all items of the quality checklist [29] and four studies contemplated more than 60% of the items [28, 30, 32, 33]. The items regarding recruitment (33.3%), sample size calculation (33.3%), and valid methods for condition identification (44.4%) were the ones which the authors failed to comply with (Table 2).

**Table 2:** Quality of the evidence—Joanna Briggs Institute’s checklist.

Author	Sample frame	Recruitment	Sample size calculation	Subjects/setting description	Sample coverage	Valid methods	Standard measurement	Statistical analysis	Response rate
Tabei et al. [28]	Unclear	No	No	No	Yes	Yes	Yes	Yes	Yes
Kwon et al. [29]	Yes	Yes	Yes	Yes	Yes	Yes	Yes	Yes	Yes
Takasusuki et al. [30]	Yes	No	Yes	Yes	No	No	Yes	Yes	Yes
Dannenberg et al. [31]	Yes	Yes	No	Yes	No	No	Yes	Yes	No
Mercadante et al. [32]	Yes	Yes	Yes	Yes	Yes	No	Yes	Yes	Yes
Oh et al. [33]	Yes	No	No	Yes	Yes	No	Yes	Yes	Yes

## Discussion

This systematic review examined the literature regarding opioid misuse in adult patients with cancer and observed that the evidence is still limited. There are few prospective studies (*n* = 6), all cross-sectional designs with high variety of misuse definitions and methodologies applied. The results revealed a wide range of opioid misuse prevalence (5.7–84.0%) and opioid misuse risk (2.4–35.4%). The pooled prevalence for opioid misuse risk was 12.3% with high heterogeneity. The analysis of the studies’ quality revealed issues in most of them, especially regarding methods of assessment.

The risk of opioid misuse seems incipient and involves great variations in reputable organisations, which have published guidelines according to the management and use of opioids in cancer pain. The European Association for Palliative Care (EAPC) [34], European Society for Medical Oncology (ESMO) [35], and American Society of Medical Oncology (ASCO) [4] have focused on recommendations providing guidance on the use of different opioids to manage cancer—or treatment-related pain in adults. The risk of opioid misuse is sparsely described [4, 34, 35]. However, the NCCN Clinical Practice Guidelines in Oncology has developed strategies to maintain patient safety and minimise the risk of opioid misuse by assessment and routinely monitoring of patients for risk factors associated with opioid misuse [36].

Previous review studies have identified enormous variability in the prevalence of opioid misuse. A narrative review reported opioid addiction prevalence among patients with cancer pain between 0 to 7.7% [37]. In addition, a meta-analysis described a pooled prevalence of 8% (1.0–20.0%) for opioid use disorder and of 23.5% (19.5–27.8%) for risk of use disorder in patients with cancer-related pain [20]. Further, a systematic review about substance use disorder in patients with cancer found a risk of substance use disorder associated with opioid use in between 18–21% in patients in palliative care [22]. In common, those reviews observed a high heterogeneity of study designs, definitions, and assessment methods. In contrast to the present study, the former reviews have included retrospectives studies and other types of articles, which may offer views from different angles, but also may increase information bias. Interestingly, retrospective studies in the last 5 years reported risk of opioid misuse between 19.6 and 39.1% [38–40], which is higher than the prevalence observed in this study, but still with some overlap.

Variation in opioid misuse prevalence can be attributed to different definitions of opioid misuse being used, variations in methodologies and assessment tools, and specific characteristics of the samples/populations studied. An example is the prevalence of opioid misuse risk, which yielded different estimates across studies using the same instrument [31–33] or when the same study compared two different instruments [32]. Likewise, one study concluded that they could not verify their findings on opioid misuse found by using an instrument followed by a clinical examination [32]. Another important aspect is the lack of reliable methods of assessment including the implementation of validated instruments. There seems to be a need for investigating the psychometric properties of the questionnaires and maybe there is a need of developing new tools.

Research in the frequency of opioid misuse in patients with cancer may not be an easy task and the challenges may comprise an array of complexities. Disease-related factors, including dropouts and refusals to participate due to compromised mental and physical conditions, may hinder accurate data collection. Moreover, the ambiguity surrounding the definition of ‘misuse’ and misuse not explicit as addiction require attention to signs and symptoms that are not so evident and trained health care professionals to identify those signs and symptoms may be needed. Systematic assessment and follow-ups on opioid management could help identifying potential misuse and sharpen the attention of the professionals. In cancer, higher age, comorbidities, cognitive impairment, and functional decline may even complicate recognition of substance misuse [41]. Furthermore, some patients wish to hide the misuse to maintain the access to the drug. Different regions and cultures may have different attitudes towards pain management and substance use, which might lead to variations in opioid misuse patterns and the reporting about them. Cultural norms regarding what is appropriate, accepted or even legal may interfere with patients trust in healthcare professionals [28].

Notably, the lack of standardised assessment instruments tailored to patients with cancer gives rise to challenges in comparing frequency rates across studies. To our knowledge only the Screener and Opioid Assessment for Patients with Pain is validated in cancer patients [39], however, the Opioid Risk Tool has also been recommended for this population observing its limitations [42]. The above-mentioned limitation was reflected in the quality assessment of the studies.

Observing that there are no clear guidelines for diagnose of opioid misuse in oncology settings, lessons from chronic non-cancer pain area prevail demonstrating that it requires a comprehensive approach combining clinical examination and assessment tools/resources to enhance the diagnosis of opioid misuse [43]. It includes observation of the patient’s behaviour (e.g., frequent requests for opioids as dose increase and earlier prescriptions, signs of intoxication, and withdrawal symptoms) and attention to family report of the patient’s behaviour indicators of opioid misuse. Moreover, systematic assessment of risk factors for development of opioid misuse and of actual opioid misuse should be a recurrent systematic task during long-term opioid treatment. Some examples of assessment were given in the analysed studies [28–33], which included structured interviews, medical judgement, and instruments for assessment of opioid misuse risk (Opioid Risk Tool and Screener and Opioid Assessment for Patients with Pain). However, other instruments may be suitable for patients in long-term opioid therapy as Pain Medication Questionnaire and Current Opioid Misuse Measure [43–45].

The present work was planned with focus on the frequency of opioid misuse in cancer with very demarcated inclusion/exclusion criteria to reduce bias, search in four important databases since inception, and analysis of study quality. However, some limitations must be acknowledged. First, we have excluded non-English written studies, which may have resulted in missing articles, especially from low- and middle-income countries. Second, only prospective studies with standardised assessment of opioid misuse were included due to fewer potential sources of bias and confounders, possibly resulting in exclusion of ‘real world/life’ studies. Third, the majority of the studies did not provide information regarding cancer stages, pain diagnoses, duration of opioid treatment, opioid daily doses, distribution of opioid drug types that could provide a more exploratory analysis. Whether these conditions may influence the frequency or risk of opioid misuse should be investigated in the future. Fourth, we could not investigate the timing of misuse start due to the study designs. Last, the quality analysis of prevalence studies has specific criteria, which may be difficult to follow in studies with convenience samples and incomplete information. We aimed to mitigate this limitation by transparently documenting procedures and adapting Joanna Briggs Institute critical appraisal checklist guideline items.

Moving forward, a consensus on opioid misuse definition is recommended in this context, as well as improvement of assessment methods, and validation of instruments to qualify diagnosis and follow-up of patients with cancer. Having in mind the tight link between a cancer diagnosis and patient’s coping mechanism, the emotional distress, anxiety, and depression are undeniable underlying psychosocial factors that can potentially trigger opioid misuse. Collaborative efforts and longitudinal studies are imperatively required to provide more comprehensive information.

## Conclusion

In conclusion, this study revealed a wide prevalence of opioid misuse between 5.7 and 84.0%, as well as a pooled prevalence of 12.3% for risk of opioid misuse. Few studies with high diversity of characteristics and high heterogeneity were found. The varied forms of opioid misuse examined, the utilisation of distinct methodologies and assessment tools, collectively may have contributed to the percentage’s disparities observed. These discrepancies underscore the need for more studies with standardised definitions, validated assessment instruments, cohesive research strategies, and well-described guidelines to accurately quantify and address this issue within patients with cancer.
